# The Progress of Dentistry

**Published:** 1875-10

**Authors:** 


### EDITORIAL. ETC.

---

*The Progress of Dentistry*—Dr. G. S. Miles, President of the Illinois State Dental Society in his annual address, at the meeting held at Ottawa, in May last, spoke as follows upon this subject:

"Dentistry, like civilization, has ever been traveling westward—from its early life on the banks of the far-famed Nile, it crossed the Mediterranean, passed successively through Greece, Italy, France, England, America. During the first struggle between Great Britain and the Colonists, one hundred years ago, a few practitioners bade farewell to the endearments of home, crossed the Atlantic and established themselves upon our eastern coast. The best information we have of the condition of Dentistry prior to its advent upon our shores is, that it was but a very insignificant calling or occupation, pursued by a few individuals without scientific attainments, whose curriculum of work was of the most limited range. Hence it is very well understood that the information possessed by the earlier practitioners of Dentistry in this country, in regard to the structure and pathology of the teeth, was very meagre and destitute of that scientiffc knowledge so desirable and essential to the successful practice at the present day. But the limited knowledge possessed by those who practiced the art at that day was only equaled by that of the people, in regard to the necessity of attending to their teeth, or the fact that any saving influence could be applied to them. The fact, also, that teeth were less subject to decay than in these latter days, occasioned a very limited demand for the services of the Dentist, and so slow was its growth, that in 1820 it is said there were but about one hundred dentists in the country.

From that period, however, to 1840 its progress was much more marked. In 1839, through the instrumentality of Harris, Brown, Hayden, Parmly and others, the *American Journal of Dental Science* was established, which, more than any one thing, exerted a greater influence in elevating the profession throughout the country. Quickly followed the establishing of the *Baltimore Dental College* by a portion of the same earnest workers, and about the same time an organization of a Dental Society national in its character, was consummated. Since these important steps were takem the advancement has kept pace with the onward march of civilization in all that ennobles a profession in its moral standing and scientific attainments, until at the present time there are some 12,000 practitioners scattered through our vast domain; half a hundred Dental Societies; ten Dental Colleges, and quite a number of journals devoted to the education of students in all branches connected with the science and art of Dentistry, the discussion of the various subjects appertaining to the profession, and the dissemination and cultivation of such ideas as may be connected with the growth of the profession and of interest to men of thought.

AlthoughDentistry was practiced in the old world to a limited extent as a *calling*, as a *profession* it is a product of this country and during the past half century.

During the past fifty years Dentistry has been slowly marching across the continent. It had passed through the rich alluvial basin of the Mississippi, crossed the Missouri and the plains beyond,—it has stood upon the high crest of the Rocky Mountains, and penetrated that vast region beneath the surface of which are riches *grander* than ancient history recounts and *vaster*,—it has swept down the western slope and made its mark in our far-famed Eldorado,—it has crossed the placid surface of the wide Pacific, and almost spanned the world. It is now looking with longing eyes across the flowery kingdom for "new worlds to conquer" and new triumphs to gain. But the worth of a profession should not be measured by its territorial expansion, but rather by its work accomplished, its deeds performed its scientific exhibit; and I take it there is a deeper interest in, and a more thorough knowledge of the practice of the profession than there has ever been before within its history. The members of the profession are beginning to investigate for themselves; to exercise their reasoning powers, instead of blindly acquiescing in the statements of others without due reflection and experiment.

Individuals may say that teeth filled fifteen to twenty-five years ago and even longer, with soft foil, are now perfectly good, while others that have been filled but a few years with cohesive gold are failing—therefore Dentistry is not progressing. But such a conclusion is fallacious, for in order to determine the relative merits of different preparations it would be well to have the same operator or grade of operators carefully observe their work through a series of years, more or less noting their condition from time to time, and by that means be enabled to determine the relative merits of different materials. Whatever may be your conclusions after patient investigation. you are farther advanced in true science and better able to perform the same character of work than ever before. Instead, therefore, of adopting a particular material because it is advertised or recommended by some one, you adopt that method of treatment and that preparation which experience and practice teaches you works out the best results. "To err is human," and Dentists do not claim perfection. I am inclined to believe that they have in the past been prone to accept the statements of others without due thought, and carry their practices to extremes in many cases; and such I believe is the case in regard to the use of cohesive gold. Operators have been made to believe that cohesive gold was the only thing to use for a good filling, and they have used it in many cases without thinking that while some operators may perform that work or fill that tooth entirely with cohesive gold, in so satisfactory a manner as to permanently save the important organ, in their hands it proves a failure; and I am inclined to think a more satisfactory result will be obtained by the employment of a larger amount of soft foil than some have been in the habit of using the past few years.

Those gentlemen who are taking an interest in conducting the number of Dental Schools in our land for the proper education of those who are entering the ranks, not only in the manipulative art but in the knowledge of cause and effect of those diseases peculiar to the oral cavity, may be the pioneers in the great work of raising the standard of our profession. Graduates of Dental Colleges ought to be men who, leaving the Institution, are able to form correct conclusions upon those subjects connected with this profession, and they, united with the large number who have not had the advantage of such instruction, but who are, nevertheless, heartily interested in building up the profession, cannot but produce a salutary effect upon the great mass of the profession in molding their professional and moral character.

The fact that so large a number of the profession are investigating and studying the principles and theories which underlie our professional fabric, is one of the surest evidences of healthy growth.

Nothing is more evident of this than the interest taken in the great number of Dental Societies throughout the length and breadth of our country, and where the members are free to express views and are bold to criticise whatever sentiments are advanced that are in opposition to their convictions, no matter from whom they eminate. This spirit of inquiry and investigation, which is more general than ever before, is a great proof of progressive earnestness in the profession, and that individuals are not allowing their enthusiasm to run away with their judgment in any particular branch, but are adopting a more conservative mode of practice.

